# Prevalence of abnormal electrocardiographic findings and associated factors among patients with epilepsy at Jimma Medical Center, Ethiopia; Hospital-based cross-sectional study

**DOI:** 10.1371/journal.pone.0301043

**Published:** 2024-05-15

**Authors:** Tadesse Dukessa Gemechu, Minase Tekabe Kasahun, Eyob Girma Abera, Kedir Negesso Tukeni

**Affiliations:** 1 Department of Internal Medicine, Jimma University, Jimma, Ethiopia; 2 Jimma University, Jimma, Ethiopia; Baruch Padeh Medical Center Poriya, ISRAEL

## Abstract

**Background:**

A person with epilepsy experiences recurrent seizures as a result of a persistent underlying disorder. About 50 million people globally are impacted by it, with 4 million of those being in Sub-Saharan Africa. One of the most frequent comorbidities that raise the mortality and morbidity rates of epileptic patients is abnormal Electrocardiographic (ECG) findings. Thus, the purpose of this study is to evaluate the prevalence of abnormal ECG findings in epileptic patients that might lead to increased risk of sudden cardiac death.

**Methodology:**

A hospital based cross-sectional study was at Jimma Medical Center of Ethiopia on epileptic patients who were on follow-up at neurologic clinics during the data collection period. The malignant ECG characteristics and was identified using the ECG abnormality tool. To facilitate analysis, the gathered data was imported into Epidata version 3.1 and exported to the SPSS version 26. The factors of abnormal ECG and sudden death risk were examined using bivariate logistic regression.

**Results:**

The study comprised 190 epileptic patients, with a mean age of 32 years. There were more men than women, making up 60.2%. A 43.2% (n = 80) frequency of ECG abnormalities was identified. According to the study, early repolarization abnormalities were the most common ECG abnormalities and increased with male sex and the length of time a person had seizures (AOR) of 4.751 and 95% CI (.273,.933), p = 0.029, compared to their female counterparts.

**Conclusion:**

The frequency of malignant ECG alterations in epileptic patients on follow-up at Jimma Medical Center in Ethiopia is described in the study. According to the study, there were significant ECG alterations in epileptic individuals. Male gender and longer duration of epilepsy raise the risk of abnormal ECG findings that could result in sudden cardiac death.

## Background

Seizures are temporary events resulting from abnormally high or synchronous neural activity in the brain, whereas epilepsy is a medical condition in which a person has a persistent underlying mechanism that increases the chance of recurring seizures [1].

Worldwide, it is predicted to afflict 50 million people without regard to sociodemographic boundaries [2]. Epilepsy is one of the most common neurological diseases, with a point frequency of 4 to 10 per 1,000 people, according to previous research [3].

According to the 2017 International League against Epilepsy classifications seizure can be classified as generalized onset, Focal Onset, Unknown and Unclassified [4]. The diagnosis of Epilepsy can be established clinically as well as with the use of EEG (electroencephalogram). Clinical diagnosis of epilepsy requires two or more unprovoked seizure occurring > 24 hours apart. Diagnosis based on EEG requires distinct EEG seizure activity during the clinically evident event (i.e., abnormal repetitive rhythmic activity having a discrete onset and termination) [5].

An electrocardiogram shows the electrical activity of the heart graphically. The commonly used and accessible 12-lead electrocardiogram (ECG) is a tool for identifying both normal and abnormal cardiac electrical activity. The identification of structural cardiac disorders is another important function of the ECG [6]. When the heart beat is recorded outside of the normal range for rhythm, rate, axis, P wave, QRS duration, PR interval, QT interval, T wave, and ST segment, it is referred to as an abnormal ECG [7, 8].

The underlying structural heart lesions or nonstructural heart disorders that might change the creation and conduction of cardiac impulses may be the cause of these aberrant ECGs. Abnormalities in electrical impulse generation, conduction, or both may also be present 4. These electrical anomalies in the seizure patients may be connected to anti-epileptic medication, the acute and long-term effects of the seizure on the heart, or potential shared risk factors for CVD. Due to the hypoxemia brought on by recurrent seizures, the cardiotoxic effects of excess catecholamines, and modifications to the autonomic nervous system and cardiac fibrosis, convulsive seizures have a pro-arrhythmic effect. Epilepsy and cardiac arrhythmia share many risk factors, such as hereditary and acquired disorders such as Long QT syndrome that impact brain and heart-derived channels [8].

For individuals with epilepsy, some arrhythmias raise the risk of sudden cardiac death. Among them are anomalies related to early repolarization, Brugada pattern, and QTc prolongation [5]. Depending on the kind of ECG irregularities the patient may be experiencing; emergency care specialists can offer the appropriate course of action in developed countries. Nevertheless, there are restrictions on the patterns of ECG alterations in seizure patients, making it difficult to provide broad strategies for managing these patient populations. Furthermore, it is uncertain how common the malignant ECG characteristics are in Ethiopian epileptic patients. Thus, the frequency of malignant ECG abnormalities that could result in sudden cardiac death and related variables of patients with epilepsy receiving follow-up care at the Jimma Medical Center’s Neurologic Clinic in Ethiopia are reported in this study.

## Methods

### Study area

Over 20 million people live in the catchment areas of Jimma Medical Center, a tertiary teaching public hospital in Jimma Town, Oromia region, Ethiopia. Over 1500 healthcare professionals work at the hospital center, providing care for over 4700 deliveries, 11,000 emergency cases, and 160,000 outpatients annually. One of the outpatient departments that handles patients with epilepsy on follow-up is the Neurologic Clinic.

### Study design and period

Between November 1, 2021, and January 30, 2022, a prospective cross-sectional hospital-based study involving epileptic patients undergoing follow-up at the Medical Center was carried out.

### Eligibility criteria

Patients with epilepsy who were at least eighteen years old, receiving treatment and follow-up during the study period, capable of submitting to ECG recording, and ready to provide informed consent to take part in the study were included. On the other hand, individuals with epilepsy who were undergoing medical ward visits, had been referred from another hospital for investigations or follow-up, were too sick to have their ECG recorded, or were unwilling to take part were not included in the study.

### Sample size determination and sampling technique

With a 95% confidence interval and a 5% margin of error, 50% of epileptic patients with potentially malignant ECG alterations were taken into account when calculating a single population proportion formula. Thus, the sample size was determined as:

$$\text{n}=[{\left(\text{Z}\alpha /2\right)}^{2}*\left({\text{P}}^{*}(1-\text{P})/{\text{d}}^{2}\right.$$

Where:

Zα/2 is the z-score for the significance (1.96 for α = 0.05)

P was the prevalence of malignant ECG changes among epileptic patients.

d = margin of error of 0.05,

n = [(1.96)^2^   (0.5   (1-05)/0.05^2^

n = 384

**Fig 1:**
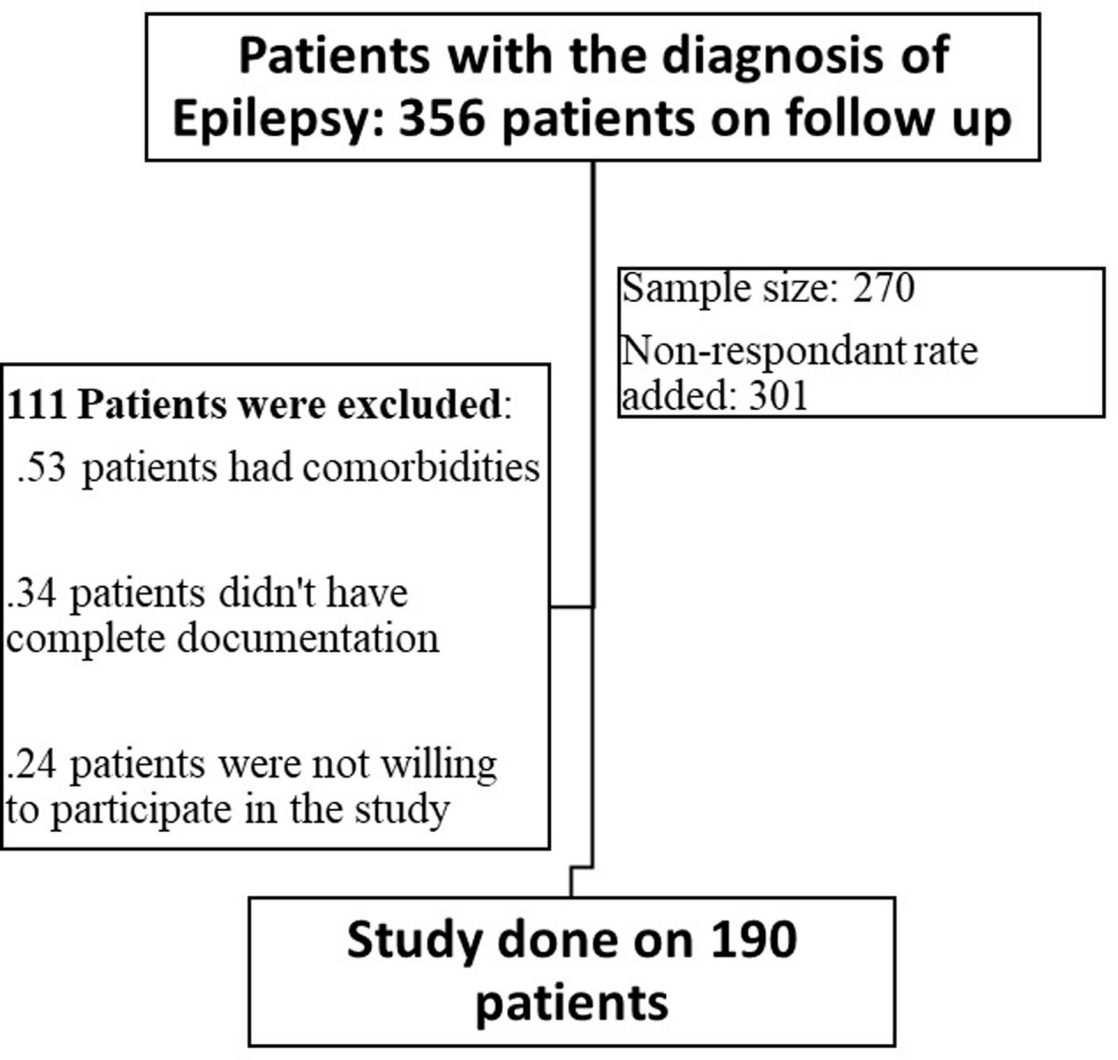
Schematic presentation of patients’ selection procedure.

356 patients were receiving follow-up care at the Neurologic clinic during the study period. Because of this, the amended sample size was 270 because the research population (n) was less than 10,000; adding 10% to the projected non-response rate made the total sample size 301. Ultimately, 190 epileptic patients who met the inclusion criteria and gave their written informed consent to take part in the trial for its duration were chosen using a straightforward random sample procedure (Fig 1).

### Data collection

Data on patients was gathered by five clinical nurse professionals who worked at the Neurologic Clinic during the study periods. A structured data collection format was used, and its correctness was checked before any data was taken. The sociodemographic details of the epileptic patients, such as age, sex, chart number, marital status, educational attainment, monthly income, and place of residence, as well as their clinical profiles, which included alterations to their ECG, were gathered. After that, the manual (adapted from Epileptic Disord, Vol. 23, No. 1, February 2021) was used to analyze the ECG alterations for each patient (Table 1).

**Table 1:** Definition of summary of common ECG abnormalities with their standard criteria.

ECG patterns	Description or definition
**Sinus rhythm**	Regular RR intervals: between 600–1000 ms where Every P wave is followed by a QRS complexes
**Tachycardia and Tachyarrhythmia (RR intervals < 600 ms)**	
**Tachycardia with narrow QRS complexes (<120 ms)**	Regular RR intervals: sinus tachycardia, atrioventricular nodal re-entry tachycardia, atrial flutter
	Irregular RR intervals: atrial fibrillation
**Tachycardia with wide QRS complexes (>120 ms**	Regular RR intervals: sinus tachycardia with pre-existing aberrant conduction
	Regular or irregular: sinus tachycardia with pre-existing aberrant conduction, RR intervals: VT, ventricular flutter, VF without organized QRS complexes
**Bradycardia and Bradyarrhythmia (RR intervals > 1000 ms)**	
**Bradycardia**	Wide QRS complexes and regular RR intervals: sinus bradycardia with pre-existing aberrant conduction, ventricular escape rhythm
	Narrow QRS complexes and irregular RR intervals: AF, sick sinus
	Narrow QRS complexes and regular RR intervals: sinus bradycardia (sick sinus, sleep), atrioventricular block
**Bradycardia Due to atrioventricular (AV) block**	2nd degree AV-block
	Type I (Wenckebach): PR interval gradually increases until one QRS complex is blocked
	Type II (Mobitz): PR interval normal, but not every P wave is followed by QRS complex (2:1, 3:1, …)
	3rd degree AV-block: complete block, AV dissociation
**Conduction interval abnormalities**	
**PR interval**	PR interval >200 ms: 1st degree AV-block
	PR interval <120 ms (plus ‘delta wave’): pre-excitation syndrome (Wolff-Parkinson White syndrome
**Wide QRS complex (> 120 ms)**	Left bundle branch block (LBBB): RsR’ in V6
	Right bundle branch block (RBBB): RsR’ in V1
	Intraventricular conduction delay: not RBBB or LBBB
**Repolarization abnormalities**	
**QT interval**	QT intervals depend on sex, age and heart rate (Need to be corrected with formulas)
**QT interval prolongation**	Corrected QT intervals (Fridericia) >465 ms in women and >457 ms in men, when heart rate 60–99 bpm
**QT interval shortening**	short QT syndromes with corrected QTc <320 ms
**QT dispersion (QTd)**	Difference between shortest/longest QT interval on 12-lead ECG
**T-wave alternans (TWA)**	Beat-to-beat alternation of morphology/amplitude of ST segment/T-wave
**ST segment abnormalities**	
**ST elevation**	Abnormal like Pattern seen ST elevation Elevated in myocardial infarction, pericarditis, left ventricular hypertrophy, early repolarization
	Infarction: Elevation>0.1 mV in limb leads or >0.2 mV in precordial leads
**ST depression**	Abnormal >0.1 mV in V5-V6, or >0.15 mV in AVF or III
**Negative T wave**	Not the same polarity as the QRS complex

### Data management and data analysis

Every day, the accuracy and completeness of the data were verified. After that, the gathered data were added to Epidata Manager version 3.1 for examination. A descriptive analysis was conducted on the study participants’ demographic characteristics. Tables are used to illustrate the analysis of categorical data, which were expressed as frequencies (n) and percentages (%). For regularly distributed data, the mean (SD) was used for analysis, and for skewed data, the median (IQR) was used. Bivariate analysis was performed using chi-square and student data, while multivariate analysis was performed using binary logistic analysis. When the cell frequency was less than five (n<5), Fischer’s exact test was used; for cell frequency (n>5), the chi-square test was employed. A significant threshold of P <0.05 was established. The correlation between the variables was displayed using the odds ratio.

### Ethical approval

The Institutional Review Board (IRB) of the Jimma Institute of Health, Jimma University, Ethiopia, granted ethical approval. Written informed consent was obtained from each participant, and all study participants and their relevant stakeholders were informed of the study’s goal. For participants who were illiterate, we employed an unbiased observant throughout the entirety of the informed consent procedure and the data collecting phase. According to the Helsinki Declaration, the study was carried out.

## Results and discussion

### Socio-demographic characteristics

The study comprised 190 patients with epilepsy in total. With a mean age of 32.2 years (SD of 11.8 years), the majority of research participants were male (60.2%; male to female ratio: 1:1.53). The majority of them were in the 20–29 age range. Thirty-four percent of the patients were unschooled. Table 2 shows that 51.8 percent of the patients were married and 51% were from rural areas.

**Table 2:** Socio demographic characteristics epilepsy patients at JUMC adult seizure follow up clinic, Jimma, Ethiopia, 2021 G.C.

*Variable*		*Frequency*	*Percent*
*Age*	*Less than or equal to 19 years*	*22*	*11*.*5%*
	*20–29 years*	*66*	*34*.*6%*
	*30–39 years*	*56*	*29*.*3%*
	*40–49 years*	*31*	*16*.*8%*
	*50–59 years*	*6*	*3*.*1%*
	*60–69 years*	*7*	*3*.*7%*
	*>70*	*2*	*1%*
*Sex*	*Male*	*115*	*60*.*2%*
	*Female*	*75*	*39*.*8%*
*Marital Status*	*Single*	*79*	*41*.*4%*
	*Married*	*98*	*51*.*8%*
	*Divorced*	*11*	*5*.*8%*
	*Widowed*	*2*	*1%*
*Educational Status*	*Illiterate*	*58*	*30*.*4%*
	*Read and write*	*9*	*4*.*7%*
	*Primary education*	*69*	*36*.*1%*
	*Secondary education*	*34*	*18*.*3%*
	*Diploma*	*11*	*5*.*8%*
	*Degree*	*8*	*4*.*2%*
	*Postgraduate*	*1*	*0*.*5%*
*Occupation*	*Government Employee*	*20*	*10*.*5%*
	*Merchant*	*10*	*5*.*2%*
	*Student*	*38*	*19*.*9%*
	*Daily Laborer*	*20*	*10*.*5%*
	*Farmer*	*52*	*27*.*2%*
	*House Wife*	*29*	*15*.*2%*
	*Other*	*21*	*11*.*5%*
*Residence*	*Urban*	*93*	*49%*
	*Rural*	*97*	*51%*

### Clinical characteristics of patients with epilepsy

Of the 190 patients with epilepsy, 160 (83.3%) had had the condition for longer than ten years. Nearly all of them were using anti-epileptic meds; the majority (62.6%) were taking one drug, while the remaining individuals were taking multiple medications to manage their epilepsy. Nonetheless, the majority of research participants—120, or 63.2 percent—still have poorly managed epilepsy. For 163 patients, or 85.9%, the cause of their epilepsy is unknown. 92.1 percent of patients did not have any other coexisting medical issues.

Ninety-seven percent of the study participants had no positive family history of epilepsy. The most frequently found etiologies of epilepsy among the remaining research participants were head trauma, brain tumors, and strokes (Table 3).

**Table 3:** Clinical characteristics of epilepsy patients at JUMC adult seizure follow up clinic, Jimma, Ethiopia, 2021 G.C.

*Clinical characteristics*	*Category*	*Frequency*	*Percent*
Age at the onset of epilepsy	<5	15	7.9%
	6–14	41	21.6%
	15–19	58	30.5%
	>20	76	40%
Duration of epilepsy	<5	52	27.4%
	6–10	44	23.2%
	>10	93	48.9%
Type of epilepsy	Generalized	164	85.9%
	Focal	27	14.1%
Taking Anti-epileptic drugs?	Yes	190	99.5%
	No	1	0.5%
	Monotherapy	115	60.2%
	Polytherapy	69	36.1%
Degree of seizure control	Controlled	70	36.8%
	Uncontrolled	120	63.2%
Comorbid medical conditions	Yes	15	7.9%
	No	176	92.1%
Etiology of Epilepsy	Known	27	14.1%
	unknown	163	85.9%
Family history of Epilepsy	Yes	14	7.3%
	No	177	92.7%
History of status epilepticus	Yes	9	4.7%
	No	182	95.3%

According to the results of the patient evaluation, the majority of the 172 patients (90.1%) had normal heart rates within the range of 60–100 bpm and normal systolic and diastolic blood pressure (mean SBP of 115 mmHg and SD of 15.1 mm HG and mean DBP of 82.2 mmHg and SD of 56.5 mm HG).

### ECG abnormalities and correlates among patients with epilepsy

Of the study subjects, 43.2% had abnormal ECG readings. Tables 4 and 5 show that early repolarization, sinus bradycardia, and long QT syndrome were the most often found aberrant ECG abnormalities. Table 6 shows that 10.5% of study participants exhibited ECG abnormalities that are known to enhance the risk of SCD.

**Table 4:** Machine generated (Rate, interval, axis and duration) ECG analysis of epilepsy patients at JUMC adult seizure follow up clinic, Jimma, Ethiopia, 2021 G.C.

ECG Findings		Frequency	Percent	Mean	SD
Rate	<60	17	6.2%	75	11.8
	60–100	165	86.4%		
	>100	2	1%		
QRS Axis	Normal	169	88.5%	59.9	59.3
	LAD	7	3.7%		
	RAD	15	7.9%		
PR interval	Normal	179	93.7%	151.5	24.8
	Short	6	3.1%		
	Prolonged	6	3.1%		
QRS duration	Normal	157	82.2%	86.06	10.5
	Wide	3	1.6%		
	Narrow	31	16.2%		
QTc interval	Normal -	189	99%	395	45.1
	Short	-	-		
	Prolonged	2	1%		

**Table 5:** Frequency of ECG specific abnormalities among epilepsy at JUMC adult seizure follow up clinic, Jimma, Ethiopia, 2021 G.C.

Abnormal ECG Pattern		*Frequency*	*Percent*
*General ECG pattern*	*Normal*	*82*	*43*.*2%*
	*Abnormal*	*108*	*56*.*8%*
Early repolarization pattern		20	10.5%
Sinus bradycardia		15	8.9%
RAD		15	8.9%
Nonspecific ST, TWAVE abnormality		13	5.3%
Sinus arrhythmia		11	5.8%
Chamber enlargement (atrial and ventricular)		8	4.2%
LAD		7	3.7%
1^st^ degree AV block		6	3%
LONG PR		6	3.2%
SHORT PR		6	3.2%
Old myocardial infarction (Q-waves)		5	2.6%
Non-specific ventricular conduction block		5	2.6%
Wide QRS		3	1.6%
PAC		2	0.5%
PVC		2	1%
Sinus tachycardia		2	1%
Prolonged QT		2	1%
Atrial fibrillation		1	0.5%
Junctional rhythm		1	0.5%

NB: One patient may have more than one abnormal ECG.

**Table 6:** ECG markers of SCD among epilepsy patients at JUMC adult seizure follow up clinic, Jimma, Ethiopia, 2021 G.C.

ECG markers of SCD	NUMBER	PERCENT
Short QT	0	0%
Long QT	2	1%
Brugada ECG	0	0%
VF/VT/WPW	0	0%
COMPLETE AV BLOCK	0	0%
ERP	20	10.5%

In the epileptic patients in this study, it was found that being male (AOR:4.751, at 95% CI:2.73,.93, P-value of 0.029) and having epilepsy for a longer period of time (AOR: 0.461, at 95% CI:0.384, 7.170, P-value of 0.497) increased the risk of having ECG abnormalities that can result in SCD (Table 7).

**Table 7:** ECG pattern changes and associated factors by bivariate analysis and chi square test among epilepsy patients.

	Group	Abnormal ECG–No (%)	Normal ECG-No (%)	Total	P-Value
Age	under 20	12 (6)	10 (5.2)	22 (11.6)	0.55
	20–39	50 (26.3)	71 (37.3)	121 (63.7)	
	40–59	15 (7.9)	23 (12.1)	38 (20)	
	above 60	5 (2.6)	4 (2.1)	9 (4.7)	
Sex	Male	56 (29.4)	58 (30.5)	114 (60))	0.02
	Female	26 (13.7)	50 (26.3)	76 (40)	
Educational Status	Formal	57 (30)	66 (34.7)	123 (64.5)	0.28
	Informal	25 (13.1)	42 (22.1)	67 (35.3)	
Residence	Urban	38 (20)	56 (29.5)	94 (49.5)	0.468
	Rural	44 (23.1)	52 (27.4)	96 (50.5)	
Age at onset of epilepsy	below 20	50 (26.3)	64 (33.7)	114 (60)	0.88
	20–39	26 (13.7)	39 (20.5)	65 (34.2)	
	40–59	5 (2.6)	5 (2.6)	10 (52.6)	
	above 60	1 (0.5)	0 (0)	1 (0.5%)	
Duration of epilepsy	< 5 years	29 (15.3)	23 (12.1)	52 (27.4)	0.03
	6–10 years	13 (6.8)	31 (16.3)	44 (23.2)	
	> 10 years	40 (20.5)	54 (28.4)	93 (48.9)	
Type of epilepsy	Focal	11 (5.8)	15 (7.9)	26 (13.7)	0.925
	Generalized	71 (37.4)	93 (48.9)	164 (86.3)	
Therapy	monotherapy	49 (25.8)	67 (35.3)	116 (61.1)	0.829
	polytherapy	32 (16.8)	41 (21.6)	73 (38.4)	
Family history of epilepsy	Yes	7 (3.7)	7 (36.8)	14 (7.4)	0.59
	No	75 (39.5)	101 (53.2)	176 (92.6)	
Status epilepticus	Yes	5 (2.6)	4 (2.1)	9 (4.7)	0.5
	No	77 (40.5)	104 (54.7)	181 (95.2)	
Comorbidity	Yes	7 (3.7)	9 (4.7)	16 (8.4)	0.96
	No	75 (39.5)	99 (53.1)	174 (91.6)	
Seizure control	Controlled	27 (14.2)	43 (22.6)	70 (36.8)	0.33
	Uncontrolled	55 (28.9)	65 (34.2)	120 (63.2)	

## Discussion

The trial comprised 190 individuals with epilepsy in total. With a male to female ratio of 1:1.53 and a mean age of 32.2 years (SD of 11.8 years), the majority of research participants were male (60.2%). Thirty-four percent of the patients had never attended school. The majority of patients (51.8%) had marital status, and 51% were from rural areas.

An abnormal ECG was found in 43.2% of research subjects. This is greater than the Iranian study that found aberrant ECG readings in 23.8% of epilepsy patients [9]. This study also revealed that abnormal ECG readings are more common in men (49.2% versus 34.2%) in women. This indicates that the proportion of malignant ECG is larger in this study than in the other study, which found that females had a higher proportion of abnormal ECGs than males [10].

The most frequent ECG abnormality found in this study was early repolarization pattern (ERP), which affected 20 individuals. Sinus bradycardia, which affected 15, and sinus arrhythmia, which affected 11, were the next most prevalent ECG abnormalities. Fifteen patients were found to have RAD. The mean QT interval was 395 ms, and the PR was 151.5 ms, which are comparable to studies conducted in the USA [9].

According to this study, 22 patients had SCD markers. Out of them, two individuals exhibited extended QTc and twenty (10.5%) showed ERP. This is less than the study conducted in The Netherlands, which revealed that 10 patients had extended QTc and 62% of epilepsy patients had ERP. However, it is equivalent to the study conducted in Egypt about the prevalence of ERP. Nevertheless, as this was a study from refractory epilepsy patients, the number might have been larger if other patient groups had been included [5, 8, 11].

Male epileptic patients exhibited a higher frequency of abnormal ECG patterns. This result is consistent with the research conducted in Japan [11]. The study found a significant correlation between male sex and the likelihood of aberrant ECG patterns, with the correlation increasing with the length of time the patient has had epilepsy. Although this result differs from that of the study conducted in Egypt, which indicated that poor seizure control and advanced age were linked to abnormal ECG changes in epilepsy patients, it is somewhat comparable to the Brazilian study that found male sex, increasing age, and polytherapy to be associated with the likelihood of having an abnormal ECG [11, 12].

## Conclusion

The frequency of ECG abnormalities in patients with epilepsy who are being followed up at Jimma Medical Center in Ethiopia is described in the study. According to the study, there were significant ECG alterations in epileptic individuals. Male gender and longer duration of epilepsy are associated with an increased risk of malignant ECG abnormalities, which may raise the risk of sudden cardiac death.

## Strength and limitation of the study

The data vacuum about ECG anomalies in epileptic individuals who may be more susceptible to sudden cardiac death in Ethiopian settings is filled in by this study. This emphasizes how crucial it is for individuals with epilepsy to undergo repeated ECG examinations. Additionally, it enables medical facilities to modernize the ways they treat epileptic patients, perhaps leading to a rise in the early identification of malignant ECG abnormalities that might be treated to lessen the chance of sudden cardiac death in candidates who are already at risk. Despite its intended intent, our study is vulnerable to observational design limitations. For example, a large portion of the data was obtained when the patients were stable, which may have led to an underrepresentation of the real ECG changes during the course of the seizure disorders. Furthermore, because there are so few data, the results cannot be extrapolated to the other patients. Our findings suggest association but not causation, as with all cross-sectional research.

## List of abbreviations

JMC: Jimma Medical Center
ECG: Electrocardiogram
EEG: Electroencephalogram
SCD: Sudden Cardiac Death
VT: Ventricular Tachycardia
VF: Ventricular Fibrillation
AF: Atrial Fibrillation
ILAE: International League Against Epilepsy
LQT: Long QT
ERP: Early Repolarization Pattern
SD: Standard Deviation
RAD: Right Axis Deviation
